# Mortality and Insanity in Separate Plan Prisons in England and America

**Published:** 1852-07-01

**Authors:** 


### MORTALITY AND INSANITY IN SEPARATE PLAN PRISONS IN ENGLAND AND AMERICA.

Dr. Parrish presented and read the following communication,* embracing the substance of the remarks made by him at the last meeting of the College, with revisions and additions.

In the *London Lancet* for June, 1851, will be found an abstract of a paper on prison discipline, read before the London Medical Society by Dr. Forbes Winslow, which, with the discussion upon it, forms matter for profitable reflection and comment.

Dr. Winslow believes, and we think with good reason, that the study of the great problem of crime and its punishment comes peculiarly within the province of physicians, who, as a class, are better qualified than any others, both from their education and pursuits, to grapple with them. With these views, Dr. Winslow appears to have carefully investigated the recent improvements in the construction and management of prisons in England, in order, if possible, to arrive at some definite conclusions as to their actual effects on the moral and physical condition of convicts.

His statistics possess unusual interest, when viewed in connexion with those obtained from the prisons of this city and county; and in this light I propose to regard them.

It is known to the fellows that the plan of separate confinement, which originated in Pennsylvania, has been partially adopted in England, as well as in some parts of the continent of Europe; and it is to this class of prisons that the inquiries of Dr. Winslow are directed. In order, however, to draw a just parallel between the separate prisons of England and our own, it will be necessary to present some of the points of difference between them; and to sketch as briefly as possible the main features of the plans now in operation in the two countries.

Our Pennsylvania system has been both highly lauded and severely condemned; and in the discussions in regard to it, a degree of acrimony and uncharitableness has been engendered akin to that which too often marks sectarian and political controversies, and which is altogether unfavourable to the development of truth. It is the province of the physician, looking at this question as a medical and philosophical one, to free himself from all undue bias, and to view it in the light of facts alone.

Regarding the origin of the system of separate (or, as it was originally denominated, solitary) confinement, all must admit that the motives of the men who projected it were eminently philanthropic. They were amongst the most benevolent and public-spirited citizens of Pennsylvania, men of pure lives and honest hearts.

Seeing the utter failure of the old and vindictive methods of punishment to reform bad men, and considering this as the paramount object of penal law, they naturally sought for something higher and better. More than a century and a half ago, the wise founder of our state had taught that the reformation of the offender should be the grand object of punishment, and that cruelty and vengeance, which had entered so largely into the penal code of the Old World, should be swept away before the advancing tide of a purer gospel. Acting upon this sentiment, the original penal code of Pennsylvania was far milder than that of the mother country, or even of the other colonies, and under the

* Stated Meeting of the College of Physicians of Philadelphia, September 2nd, 1851. Dr. Meigs, Vice-President, in the chair.

mild sway of Penn, prisons were houses of correction rather than bastiles for executing vengeance. Nor was the example of the founder wholly lost upon succeeding generations. What was then rather a peculiar idea is now the popular sentiment in the most enlightened states.

It was in furtherance of the grand idea of reformation and correction of offenders that the plan of separating them from each other during their incarceration was conceived and carried out. The bad influence of their promiscuous intercourse in crowded rooms and workshops had been abundantly verified. It presented, in fact, an insurmountable obstacle to reformation, and was, in many instances, a direct cause of increased moral contamination, especially to the young and uninitiated.

To counteract this, entire separation, both by day and night, was offered as a substitute. It was believed that solitude would furnish a strong incentive to moral renovation. That the convict, shut out from society, with every motive to deception and to the indulgence of his passions taken away, without external objects of interest or amusement upon which his mind could rest, with no books but those of a serious kind, and deprived of the knowledge of the stirring events which were transpiring in the busy world around him, his thoughts would necessarily turn to the errors and crimes of his past life, which, as he looked back upon them, would lead him to deep contrition, and it might be to sincere penitence.

It was believed, too, that, by exclusion from the public gaze, and from his fellow-prisoners, he might emerge from his solitary cell, at the expiration of his term, and go out into the world without having the brand of the convict fixed upon him, and thus be enabled to commence anew the struggles of life. Plots of mischief and rapine, laid within the prison walls, to be executed by trained bands, grown old in crime, after their discharge, would thus be prevented, and the public security be thereby promoted.

These were some of the considerations which impelled many of our fellow-citizens to give their hearty support and co-operation to a new and then untried method of prison discipline. The plan seemed feasible: it held out the flattering prospect of reforming vicious and dangerous men, not by bayonets, stripes, shower baths, or other physical torture, but by the gradual softening influence of solitude, combined with moral and literary instruction. The early advocates of the system were divided on the question of introducing labour into the prison, some of them even supposing that occupation might amuse and distract the mind, and thus lead it off from serious reflections; but this idea was overruled by the more sagacious, and daily labour was, from the first, made a part of the scheme. Different opinions prevailed also in regard to the amount of intercourse which should be permitted between the prisoners and their officers, visitors, &c., as well as upon some other details of the plan.

*The separation and non-intercourse of the prisoners, under any circumstances,* were, however, agreed upon by all parties, and constituted the cardinal feature of the system. This was made the basis of a revised penal code, which was adopted by the legislature at its session in 1829, and has since been the law of the Commonwealth. Each prisoner is now sentenced to "solitary or separate confinement at labour" for a term corresponding with the gravity of his offence, or of the circumstances under which it was committed.

The introduction of this system involved, of course, the erection of new and costly buildings adapted to the end in view. The chief object being to isolate each occupant from his fellows, separate cells were constructed, facing upon lofty arched corridors, which radiated from a central hall. These cells are separated from each other by massive walls 14 feet long and 7½ wide; the light is admitted from above, through a skylight 22 inches long by 4 in width, and ventilation is effected by an opening in the top of the cell, which may be opened or closed at the discretion of the prisoner.

A cess pipe occupies a corner of the apartment; and across it pass two iron rods for the conveyance of heat. A double door, the inner of firm iron grating, and the outer of thick wood, guards the front and rear opening of the cell. The floors of most of the cells are of plank upon a firm joice laid on cement, while some others are of stone. To each cell is attached a yard of the same size, enclosed by a massive wall 11 feet high, to prevent intercourse during the hour devoted to exercise.

A room thus constructed, without open doors or windows, and surrounded by a wall 11 feet high, with no other opening for sun light than through a narrow skylight at the top, and with imperfect means of ventilation, and this in a great measure under the control of its occupant, must be somewhat damp, and oftentimes offensive, especially when used alike for work, eating, sleeping, and, in fact, for all other purposes. In this apartment the prisoner is strictly confined at labour, one hour of each day being allowed for exercise in the cell yard, unless evidences of ill health or mental disorder induce the physician to direct a relaxation of the discipline.

Each block contains about 34 cells on each side of the hall, and is under the care of an overseer, who is obliged to inspect the condition of each prisoner at least three times daily, and to superintend his work. The warden, overseers, physicians, official visitors, and members of the acting committee of the Prison Society are the only persons allowed to hold intercourse with the prisoners. Until recently, the principal trades pursued in these cells were weaving, shoe-making, and bobbin winding.

Without entering farther into the details of the separate system, it may be remarked that it was carried into effect in Pennsylvania, under the most flattering auspices, in the year 1829, and has since been in full operation in the state prisons at Philadelphia and at Pittsburg, and more recently in several of the county prisons in the interior. The prison at Philadelphia was opened in the 10th month (October), 1829, and soon attracted a large share of attention from philanthropists, statesmen, jurists, and from the public generally. The order, decorum, and quiet which reigned within its walls; the absence of the sad spectacle of human depravity and wretchedness, which meets the eye in the thronged apartments of prisons conducted on the old plan, excited general admiration. Commissioners from England and France were sent out to visit it, and returned to their respective countries with the most favourable reports.

These evidences of regard naturally excited a degree of state pride, and induced Pennsylvanians generally to feel themselves identified with a movement which originated in their State, and which promised to effect a most desirable reform in penal law over the world.

If any had doubts of the harmony of the plan with the laws which govern the human frame; or imagined that close and long-continued confinement in cells, such as have been described, would breed disease and death; or that in strict seclusion from society the mind would feed upon its own thoughts until it became morbid and deranged—they silenced their fears, and determined to await the results of experience.

The institution was placed under the charge of five inspectors. It was officered with an efficient and humane body of men, fully impressed with the importance of the experiment upon which the State was about to enter, and as its medical attendant, a gentleman was selected whose experience as physician to the old Walnut-street prison, and whose high character, both morally and professionally, offered the best security that nothing would be left undone to secure to the inmates as good a degree of health as was compatible with their position and circumstances. For the first few years nothing transpired to excite doubts in the propriety or humanity of the plan of separate confinement. The medical reports, though written with great caution and accuracy, bore testimony to the general good health of the prisoners, and to the safety of the course to which they were subjected.

As, however, the number of persons brought under the discipline of the institution increased, and the influence of long periods of imprisonment became more evident, facts accumulated which appeared to place a different aspect on the question.

The observations of our esteemed fellow, Dr. B. H. Coates, who, as a member of the Prison Discipline Society, was a frequent visitor at the institution, induced him to believe that there was a large mortality from scrofula and consumption amongst the prisoners of African descent; and on investigating the subject with his usual candour and accuracy, he proved conclusively that separate confinement was particularly injurious to the coloured race. Dr. Coates' paper was published in 1843, although his first communication on the subject was made to the Prison Society in 1840. In addition to this, a large number of cases of acute dementia began to appear in the medical reports of Dr. Darrach during the years 1839, 1840, and 1841, occurring more frequently amongst the coloured prisoners, and believed by Dr. Darrach to be mainly attributable to self-abuse.

Without proceeding farther with this history, we shall endeavour to lay before the College a brief summary of the sanitary condition of this institution, together with that of the county prison at Moyamensing, down to a recent period, and then proceed to a comparison between them and the English prisons.

The whole number of prisoners received at the Eastern State Penitentiary, to the close of the year 1848 (as contained in the annual report for that year), is 2421, of whom 1631 were white, and 790 coloured. The number of deaths recorded to this time, embracing a period of 19 years, is 214, or nearly 90 in the 1000, or within a fraction of 9 per cent. of the whole number received. Calculating the mortality of each year from the average number of convicts for the year, and then giving the average annual mortality for the whole period, this per centage would be considerably reduced; but the former method appears to me to convey a more accurate idea of the relative proportion between the number subjected to confinement and the number of deaths, besides being that applied by Dr. Winslow to the English prisons, with which we shall presently compare them.

The class of prisoners received at this institution is not of the worst description, nearly two-thirds of them coming from the rural districts; thus, from 2176, received to the close of the year 1846, 948 were from Philadelphia County, and 1228 from the country; of 124 new prisoners received in 1847, 43 from Philadelphia County, and the remainder from the country; and of 121 received in 1848, 40 were from Philadelphia, and the rest from the other counties.

The sentences of the convicts range from one to twenty-one years, their average duration being about three years.

The accustomed population of the prison for some years past has been about 300. The deaths in any one year vary from 1 to 26, and are generally from chronic disorders, scrofula and consumption being the most prominent. Acute diseases, and especially infectious and epidemic disorders of a low type which so frequently scourge crowded and filthy prisons, are unknown at Cherry Hill. The most fruitful source of the large mortality indicated by the above figures is from the deaths amongst the coloured prisoners; from the 790 coloured inmates received to the close of the year 1848, 141 deaths occurred, being nearly 18 per cent. of the whole number.

It would be easy to give the annual average mortality of this class, as derived from the average number in prison in each year. Thus, in looking through the reports, I find in two years, 1830 and 1833, there were no deaths amongst the coloured, while in 1831, the average mortality of this class to the average number in prison was 10·03; in 1832, 13·52; in 1834, 6·68; in 1835, 4·61; in 1836, 6·74; in 1837, 6·49; in 1838, 11·80, &c.; while, according to Dr. Emerson's tables, the annual average mortality of the coloured population of Philadelphia, of both sexes and all ages, between the years 1830 and 1840, was about $3\frac{1}{4}$ per cent.

(*To be continued.*)

